# The clues offered by SNAREs on the vacuoles of plants and animals

**DOI:** 10.3389/fpls.2025.1599323

**Published:** 2025-06-23

**Authors:** Fabrizio Barozzi, Miguel Sampaio, Cláudia Pereira, Gian Pietro Di Sansebastiano

**Affiliations:** ^1^ DiSTeBA (Department of Biological and Environmental Sciences and Technologies), University of Salento, Lecce, Italy; ^2^ GreenUPorto – Sustainable Agrifood Production Research Centre/Inov4Agro, Department of Biology, Faculty of Sciences, University of Porto, Porto, Portugal

**Keywords:** vacuoles, LROs, melanocytes, adipocytes, lipid droplets, SNAREs

## Abstract

Vacuoles and lysosome-related organelles (LROs) are essential compartments in eukaryotic cells playing crucial roles in storage, degradation, signaling, and homeostasis. Despite their functional similarities, these organelles have traditionally been studied in isolation within plant and animal cell biology. This review bridges these disciplines by exploring the molecular parallels between plant vacuoles and animal LROs, with a particular emphasis on the SNARE (Soluble N-ethylmaleimide-sensitive factor Attachment Protein Receptor) protein family, which governs membrane fusion and trafficking. SNARE complexes orchestrate intracellular transport ensuring the correct delivery of cargo to vacuoles and LROs. By analyzing SNARE homologs and their interactions across kingdoms, we highlight conserved mechanisms that regulate organelle biogenesis, remodeling, and function. This comparative approach not only advances our understanding of cellular compartmentalization but also sheds light on potential applications in biotechnology, stress adaptation, and human disease research. Integrating knowledge from plant and animal systems offers a powerful framework for discovering novel regulatory pathways in membrane trafficking and cellular homeostasis.

## 1 Introduction

A simplified narration state that “a vacuole is a membrane-bound cell organelle. In animal cells, vacuoles are generally small and help sequester waste products. In plant cells, vacuoles help maintain water balance. Sometimes a single vacuole can take up most of the interior space of the plant cell.” This citation was taken from the very popular NIH website (https://www.genome.gov/genetics-glossary/Vacuole) and leads to the misbelief that vacuoles are a plant-related biological topic. Of course, specialists of adipocytes and melanocytes know it is not the case, but it becomes a problem for young researchers building up their background and causes failing in stimulating more comparative research on this fundamental compartment.

**Figure 1:**
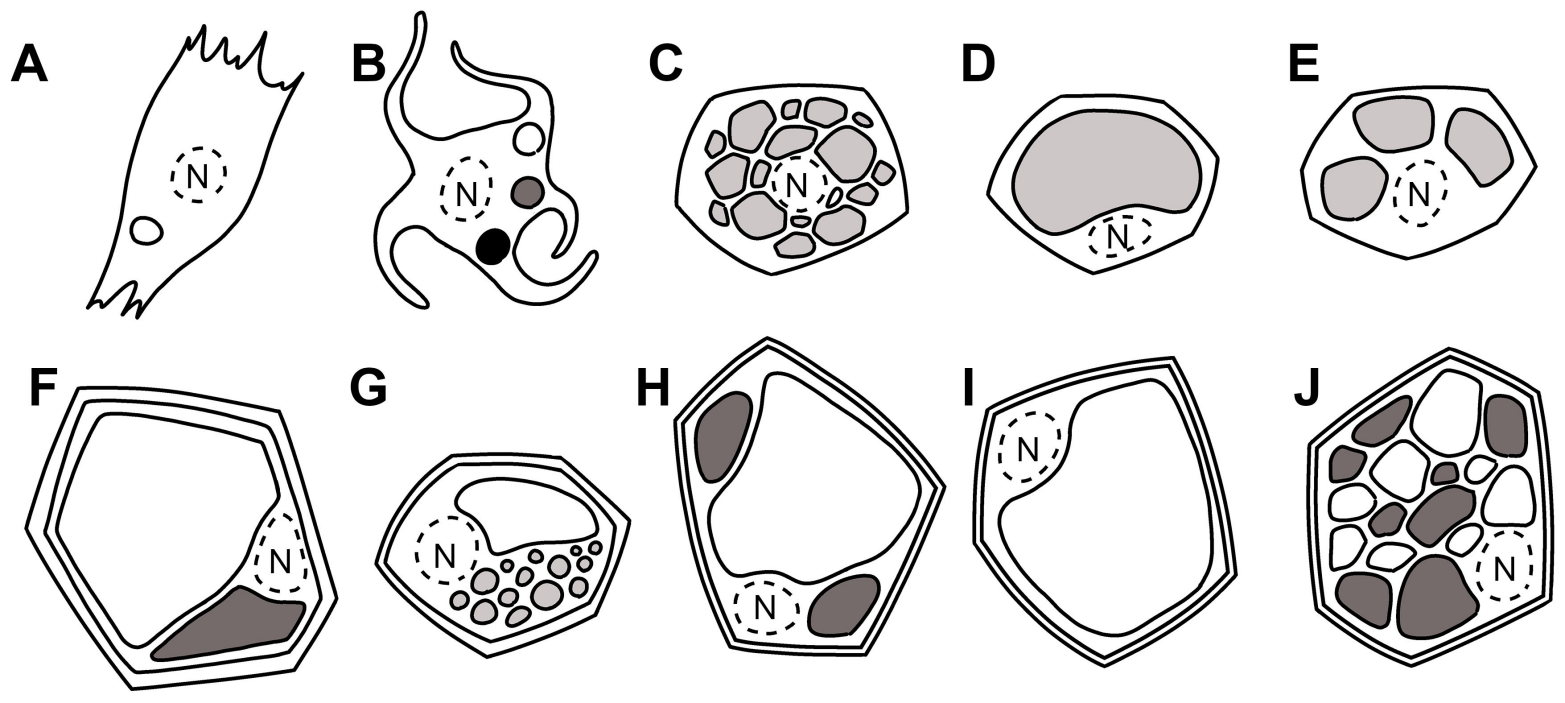
Simplified drawing of eukaryotic cells and their vacuolar content. Nuclear space is limited by a dotted line. **(A)** Melanocyte in pigmented animals’ skin (Yamashita et al., 2005) with melanosomes in the three stages differentiated by gray intensities, **(B)** isolated mammalian cell, **(C)** brown adipocytes with lipids in many small droplets (multilocularity), **(D)** white adipocytes with lipids in a unique vacuole (unilocularity), **(E)** beige adipocyte, **(F)** plant cell with tannin-rich and tannin-less vacuoles (Fleurat-Lessard et al., 2016), **(G)** central vacuole and lipid droplets, **(H)** central vacuole and small vacuoles, **(I)** typical central vacuole, **(J)** multiple vacuoles in meristematic plant cells.

Vacuoles in animal cells are considered lysosome-related organelles (LRO) but recall the fate of plant vacuoles since the specific physiological functions of each LRO is related to cell type-specific composition of their membrane. As conventional lysosomes (**Figure 1A**), LROs have often low pH at some maturation stage. Several proteins are present in these compartments as in lysosomes, but they also have unique components and functions (Bowman et al., 2019; Delevoye et al., 2019; Watts, 2022). LROs are part of the endomembrane system undergoing remodeling and intense traffic. Traffic specificity is mediated by interactions between SNAREs (soluble N-ethylmaleimide-sensitive factor attachment protein receptors) (Gu et al., 2020), and it is plausible that SNAREs play a crucial role in vacuolar biogenesis in animals as in plants, but research on animal vacuoles did not reveal much on this specific topic. The scope of this review is to evidence analogies between animal and plant vacuole biology to promote the study of dedicated literature to two researcher communities that rarely cross paths. The key element used to create connections will be the gene family of SNAREs.

SNAREs are characterized by a highly conserved coiled-coil region, defined SNARE motif, consisting of 60–70 amino acids arranged in heptad repeats. At the C-terminus, SNAREs have a hydrophobic transmembrane domain, connected to the SNARE motif through a linker region, while at the N-terminal extremity, many SNAREs contain a domain that regulates diverse functions. Initially, SNAREs were divided into two groups as follows: v-SNAREs associated with the donor vesicle membrane and t-SNAREs associated with the target organelle (Hong, 2005). Subsequently, they were reclassified into R-SNAREs (with the SNARE motif containing an arginine residue), located on the vesicle membrane, and Q-SNAREs (with the SNARE motif containing a glutamine residue), located on the target compartment and further divided into three groups (Qa-, Qb-, and Qc-SNAREs). During the docking and fusion process, three Q-SNAREs on the target membrane assemble and interact with an R-SNARE on the donor compartment forming the trans-SNARE complex constituted by four α-helices provided by each SNARE motif. After fusion, the trans-SNARE complex becomes a cis-SNARE complex that is disassembled by α-SNAP (soluble N-ethylmaleimeide-sensitive factor attachment protein) and NSF (N-ethylmaleimeide-sensitive factor) and subsequently recycled (Brown and Pfeffer, 2010).

Being responsible for membrane traffic specificity, SNAREs are key elements in membrane biology. The phylogenetic trees show that many SNARE subclades, including plants’ Qa SYP1, Qc USE11-12, and Qc SYP51-52, appeared during the evolution of multicellular land plants (Embryophyta), with a similar story for syntaxins (STX) in higher animals (Sanderfoot, 2007). These gene subclades of more recent evolution contain domains involved in additional protein–protein interactions supporting an increased complexity of inter- and intracellular communication systems associated with multicellularity.

SNARE complexes functioning in the late endosomes and vacuole/lysosomes may be very lineage specific. Most of the fungi have one dedicated Qa-SNARE, but yeast has the following two functionally distinct Qa-SNAREs: Pep12p on the late endosome/prevacuolar compartment and Vam3p on the vacuole (Sanderfoot, 2007). Mammals also have two Qa-SNAREs functioning between the lysosomes and endosomes, syntaxin 7 and syntaxin 13 (Mullock et al., 2000; Sun et al., 2003). Plants often have multiple genes in the SYP2 family showing functional differences. For example, studies on *Arabidopsis* have shown that SYP21 preferentially works on the late endosomes, while SYP22 works on the vacuoles (Rojo et al., 2003; Yano et al., 2003). Both SYP2 interact with the same partners Qb-VTI11 and Qc-SYP5 (homologous to mammalian syntaxin 8) being able to distinguish between Qb-VTI11 and -VTI12 as also shown by Qa-SYP41 (Sanderfoot et al., 2001). Members of the R-VAMP71 clade have been shown to localize to the vacuolar membrane (Carter et al., 2004; Uemura et al., 2004) suggesting that these may also be involved with vacuolar/late endosomal trafficking similar to some of the roles indicated for VAMP7 in mammals (Pryor et al., 2008; Ohbayashi and Fukuda, 2018).

The possibility that SNAREs have additional structural roles was also investigated since most of them are present in excess and concentrated in clusters, thus constituting a spare pool not readily available for interactions. Among the first evidence, there is an observation that SNARE silencing by siRNA induced an enhanced docking instead of the expected inhibition (Bethani et al., 2009). It seems that SNARE concentration is inversely proportional to the expected fusogenic activity (De Benedictis et al., 2013). These have been proposed to belong to a new functional class called interfering SNAREs or i-SNAREs (Varlamov et al., 2004). The i-SNAREs would inhibit fusion by substituting for, or binding to, a subunit of a fusogenic SNARE pin to form a non fusogenic complex.

Even earlier, Varlamov and co-workers (2004) suggested that non-fusogenic SNARE complexes, including the i-SNARE partners, have a physiological function at the level of the Golgi apparatus to increase the polarity of this organelle. This would ensure that ER-derived vesicles fuse with the cis-Golgi, while retrograde transport vesicles from endosomes fuse to the trans-Golgi.

The functioning of i-SNAREs (hsSyntaxin6/Tlg1, GS15/Sft1, ScrBet1/Bet1, AtSYP2/SYP5) in mammals, yeasts, and plants is conserved (Di Sansebastiano, 2013). SNARE accumulation outside their fusogenic active complexes seems to have a structural role in the maintenance of membrane identity and may be used to draw analogies between sorting mechanisms occurring in very diverse organisms’ cells (**Figure 1**). This is not a mere exercise, as the comparison will help trace pathological aberrations or unknown processes for macromolecule accumulation, among the multiple possibilities observable in nature thanks to essentially conserved molecular mechanisms.

## 2 Melanocyte’s vacuoles

Melanocytes are “pigment cells” (**Figure 1B**) originating from the neuroectoderm (**Figure 1A**). They are not restricted to cutaneous locations but are present in different anatomical regions, including the stria vascularis of the cochlea, the substantia nigra, leptomeninges, locus coeruleus within the brain, cardiac tissue, and adipose being “associated” with other tissues, including the nerves. Melanocytes, to become pigmented, generate melanin within initially non-pigmented vacuoles named melanosomes (Cui and Man, 2023). Melanosomes are subcellular organelles, well visible in epidermal and follicular melanocytes, and also in developing pigment cells of the eye.

As in plant vacuoles, melanosomes’ components must be sorted, delivered, and targeted specifically. Moreover, some of the organelle’s content must stay separated until the final destination and are sorted separately, for instance, to prevent generation of oxidative melanin intermediates (Le et al., 2021). Melanosomes within cells that synthesize the black and brown eumelanins develop through four morphologically distinct stages. Melanosomes in melanocytes that make red and yellow pheomelanins have a distinct structure but have not been well characterized.

Stage I melanosomes correspond to conventional vacuolar early endosomal compartments: round organelles integrated into the endocytic pathway with an electron-lucent lumen displaying a planar clathrin coat and intraluminal vesicles (ILVs). These can also be considered early endosomes (Le et al., 2021), but in melanocytes and developing eye pigment cells, these organelles evolve, since their ILVs contribute to the formation of unusual fibrillar structures. These structures elongate and assemble into fibrillar sheets as the compartment mature into Stage II melanosomes (Le et al., 2021). Stage II melanosomes contain parallel or concentric proteinaceous fibrils able to physically distend the shape of the compartment into an ellipsoidal organelle. Anyhow, Stages I and II can be indicated as “premelanosomes” since they are unpigmented organelles. The fibrils’ structure serves as template for the deposition of melanins after synthesis and guide the maturation of the organelle in Stages III and IV pigmented melanosomes (Benito-Martínez et al., 2020).

Stage I melanosomes are intermediate compartments in the endocytic pathway accessible to endocytosed tracers. Starting from Stage II, melanosomes are no longer accessible. Stages I and II melanosomes are acidified by the proton-importing activity of the ubiquitously expressed vacuolar-type H(+)-ATPase (V-ATPase; (Tabata et al., 2008). Then, Stage II melanosome and endolysosomal maturation diverge. Melanocytes continue to produce both maturating melanosomes and an active late endolysosomal pathway. The Stage I melanosome/early endosome seems to be a differentiation hub (Raposo et al., 2001). It is also possible that distinct subpopulations of early endosomes co-exist, one giving rise to the LRO and the other to late endosomes/lysosomes (Le et al., 2021). The co-existence of the two pathways ensures that melanocytes maintain both pigmentation and degradative/metabolic capacities. How Stage I melanosomes transform into both Stage II melanosomes and late endosomes has not been clearly elucidated (Le et al., 2021).

Melanosome structure and function are defined by many specific resident transmembrane proteins. Related genetic defects cause various forms of hypopigmentation of the hair, skin, and eyes up to visual impairment; therefore, the discovery of all traffic and functioning mechanisms are essential.

Mature melanosome pH regulation occurs thanks to the protein OCA type 2 (OCA2, also known as p-protein), a Cl-selective ion channel (Le et al., 2021). OCA2 resides in its active form on the melanosome membrane for a short time rapidly associating with invaginating membranes of newly forming ILVs. Its sorting into ILVs requires cargo ubiquitylation, followed by its recognition by ESCRT-0, -I, -II, and -III (Gruenberg, 2020), but its sorting to ILVs can also be ubiquitin/ESCRT independent. Moreover, OCA2 is detected on only a subset of melanosomes, and its trafficking is not yet clear (Le et al., 2021).

Stage I melanosomes are the source of cargoes delivered to maturing Stage III melanosomes, but an additional traffic pathway originates from the Golgi apparatus/TGN to contribute to melanin accumulation in Stage III melanosome by the delivery, through vesicles, of specific markers (DCT and MART-1 (Patwardhan et al., 2017)). This indicates that maturing melanosomes receive material from both endocytic and exocytic traffic. Two enzymes for melanin synthesis [tyrosinase (TYR); dopachrome tautomerase (DCT)] follow a different sorting pathway to maturing melanosomes from that of a third one [tyrosinase-related protein-1 (TYRP1)] indicating that only when these three pathways converge, pigmentation occurs. The existence of distinct sorting pathways suggests that melanosomal components must remain physically separated before the arrival in melanosomes, probably to prevent oxidation of melanin intermediates.

Recognition of transmembrane proteins is triggered by the binding of specific oligopeptidic signals on membrane cytosolic face by adaptor proteins (APs) on specific membranes (Sanger et al., 2019). AP-2 functions at the plasma membrane to sort cargoes for endocytosis, whereas AP-1 and AP-3 function on endosomes and at the TGN as also observed in plants (Liu et al., 2022 and literature within). The clearest role for APs in cargo sorting to melanosomes is for AP-3 (Dell’Angelica et al., 1999), but each AP may define a different, potentially overlapping, trafficking mechanism. AP-3 is required for OCA2 trafficking, while AP-1 controls TYRP1 traffic (Huizing et al., 2001; Theos et al., 2005). RAB6 could also be involved with the AP-mediated trafficking. The RAB6 effector ELKS guides the docking and fusion of Golgi-derived vesicles to the plasma membrane (Grigoriev et al., 2007, 2011) but also controls RAB6 vesicles targeting pigmented melanosomes (Patwardhan et al., 2017).

For targeting cargoes to melanosomes, membrane carriers must fuse with the melanosomes. One of the pathways depends on BLOC-1, an eight-subunit complex involved in cargo transport from endosomes to maturing melanosomes. The v-SNARE in this traffic step seems to be VAMP7, a SNARE protein that mediates fusion within the late endosomal/lysosomal and autophagy pathways in other cell types. It is necessary for TYRP1 targeting (Jani et al., 2015; Dennis et al., 2016), and GFP-tagged VAMP7 was localized on melanosomes (Dennis et al., 2016). The specific t-SNAREs partner of VAMP7 may be different. Syntaxin-13 (a.k.a. syntaxin-12) is needed for pigmentation and TYRP1 trafficking (Jani et al., 2015) and binds *in vitro* to BLOC-1 (Huang et al., 1999; Moriyama and Bonifacino, 2002; Ghiani et al., 2010), but it is not localized on melanosomes (Dennis et al., 2015; Delevoye et al., 2016). This SNARE localizes to the plasma membrane at steady state and is thus unlikely to function as the melanosomal t-SNARE, but it was anyhow found to form a complex with VAMP7 in endosomes (Le et al., 2021). A number of SNARE proteins are upregulated in mice during melanoma differentiation into a pigmented phenotype (Wade et al., 2001), but only VAMP7 has been directly related to melanosome targeting.

Like other organelles, melanosomes must control their size to maintain homeostasis and function. This implies that the anterograde flow of components to melanosome is coupled with a retrograde transport. The VAMP7 dynamically associates with pigmented melanosomes and is exported in tubular transport carriers that are distinct from those involved in its anterograde traffic (Dennis et al., 2016). In fact, these melanosome-derived tubules do not have classical melanosomal and endosomal markers suggesting that they are used to retrieve the unwanted contents from melanosome, including VAMP7 (Dennis et al., 2016).

## 3 Adipocyte vacuoles

Adipocyte vacuoles may be considered “elusive compartments.” An interesting review underlines how these compartments took a long time to be considered as real compartments. Initially, it was shown that TAG in lipidic monolayers might be directly transferred from mitochondria to the vacuole, then characteristic proteins were identified on their surface indicating that they may be compartments with a clear identity (Coleman, 2020). By their characteristics, technically they are not vacuoles but lipid bodies as those observed in plant cells. The important aspect we want to evidence is that, despite their origin similar to lipid bodies and oleosomes, they are regulated in their different arrangement in different cell kinds, similar to plant vacuoles.

For example, brown and white adipocytes are morphologically different. At light microscopy level (Malide, 2001), brown adipocytes have cytoplasmic lipids arranged as numerous small droplets (multilocularity, **Figure 1C**). They are rich in mitochondria and specialized in thermogenesis. White adipocytes have cytoplasmic lipids arranged in a unique vacuole (unilocularity, **Figure 1D**), and its main function is energy storage and secretion of hormones and cytokines to regulate metabolism (Puche-Juarez et al., 2023). A third type of adipose tissue has been described as “beige” (Nedergaard et al., 2007). It is halfway between brown and white adipocytes (**Figure 1E**), with a unique gene expression pattern.

Brown and white adipocyte differentiation process shows distinctive features, but in some conditions, white and brown adipocytes can interconvert. These cytotype interconversion can change metabolic needs and have great importance in physiopathology of obesity (Cinti, 2002). The most accredited theory is that there is no common precursor for white and brown preadipocytes. Brown preadipocytes have a “myogenic signature.” Brown adipocytes immersed in white tissue are “beige adiopocytes” and appear to come from a different precursor than adipocytes in brown tissue (Wu et al., 2012). In these cells, both ER and Golgi apparatus seem to contribute to vacuole differentiation, but the proteins involved in traffic regulation have been poorly investigated. In *Drosophila melanogaster*, lipid storage depends on Rab32, which was shown to localize mainly to the ER membrane (Ao et al., 2014). Rab32 is also known to be involved in forming autophagic vacuoles and in the biogenesis of melanosomes (see next paragraph) in skin melanocytes (Fukuda, 2021).

Keeping the origin of white and brown adipocytes separated, and accepting their different vacuolarization, a question must be asked. How can small vacuoles in brown cells remain separated despite membrane affinity? An important role in this could be played by SNAREs, but not much is known about them in these cells. It is simply accepted that SNARE proteins mediate lipid droplet (LD) fusion. It was demonstrated that the SNARE complex, syntaxin 18 (STX18)–SNAP23 (synaptosomal-associated protein of 23 kDa)—SEC22B, drives LD fusion in adiposome lipid mixing and content mixing *in vitro* assays (Fu et al., 2023). A previous study identified a different complex, formed by syntaxin-5 (STX5)—SNAP23 and VAMP4 (vesicle-associated membrane protein 4). The discovery of different SNARE complexes involved may indicate the possibility of different membrane identity and specific compartmentalization.

Moreover, a few SNAREs known to be present on animal vacuoles are also involved in other membrane traffic steps, such as Sec22b, which controls traffic of ERGIC (ER and ER–Golgi intermediate compartment) proteins to phagosomes (Cebrian et al., 2011). VAMP4, interacting with a number of SNAREs associated with the trans-Golgi network (TGN) and the endosome, connects traffic between the exocytosis and endocytosis. Their presence in the vacuole membrane may indicate a different contribution from donor compartments.

## 4 Plant cell vacuoles

Vacuoles are the largest, generally acidic, compartments in plant cells occupying up to 90% of the cell volume. They store nutrients and metabolites and also helps maintain turgor pressure—a unique requirement for plant cell rigidity and growth (Fluckiger, 2003; Pereira and Di Sansebastiano, 2021). They are classified according to their functions as protein storage vacuoles (PSV) and lytic vacuoles (LVs). PSVs are abundant in seed and storage tissues storing proteins and amino acids crucial for germination and seedling development (Fluckiger, 2003). LVs, on the other hand, have a degradative function very similar to lysosomes in animal cells housing hydrolases for breaking down cellular components during senescence or stress conditions (Cao et al., 2020). These two types of vacuoles can often coexist in the same cell (**Figures 1F–H**), especially during seed germination, facilitating rapid adaptation to nutrient availability and growth requirements (Tan et al., 2019) and persist in a form with neutral pH making the plant vacuolar system quite complex (Fluckiger, 2003). Senescence-associated vacuoles (SAV) (Carrión et al., 2014) or NaCl-accumulating vacuoles (Epimashko et al., 2006) were also described. Compartments of the vacuolar system rearrange depending on the type of cell/organ and its function within the plant. Co-existing LVs and PSVs can be found in cardoon flower cells of the pistil (Pissarra et al., 2007), in some varieties of petunia petal epidermis cells (Faraco et al., 2017), *Mimosa pudica* motor cells (Fleurat-Lessard et al., 1997), and a few more.

Recent experimental evidence (Hachez et al., 2014) suggests that several classes of proteins associated with vacuolar functions, such as aquaporins, ATPase pumps, channels, or SNARES, control specific events of membrane transport leading to important events of vacuolar reorganization under adverse environmental conditions. Up to now, vacuoles have been better characterized by specific types of aquaporins (AQPs), the tonoplast intrinsic proteins (TIPs). γ-TIP (TIP1) usually labels the lytic vacuole, and α-TIP (TIP3) labels the protein storage vacuole (Vitale and Raikhel, 1999). Of course, the situation is more complicated since overlap of markers has been observed in vacuolar remodeling during the transition between distinct types of vacuoles (Olbrich et al., 2007; Bolte et al., 2011). Moreover, SNAREs and AQPs can interact (Jones et al., 2014) with potentially strong effects on vacuolarization (Barozzi et al., 2019).

The studies conducted in plant cells often evidence an incomplete colocalization of tonoplast proteins, such as receptors (VSR and RMR), SNAREs, proton pumps (V-ATPase, V-PPase), and transporters (ABC, MATE), suggesting that mechanisms active in vacuole biogenesis cross paths with the vesicular trafficking of the differentiated cell generating a continuous endomembrane maturation process (Pereira and Di Sansebastiano, 2021).

As evidenced for animal vacuoles, the vacuoles in plants also receive membrane from the ER and also from the Golgi and TGN. More recently, the two TGN-associated members of the EPSIN gene family, EPSIN1 and MTV1 (modified transport to the vacuole 1), have been used to underline the presence of spatially and functionally separated subdomains of TGN in plant cells (Heinze et al., 2020) involved either in secretory or vacuolar trafficking (Shimizu et al., 2021).

TGN subdomains may also differentiate, for the contribution to different membrane traffic routes, from the endoplasmic reticulum (ER), in particular (De Caroli et al., 2020). Direct traffic from the ER to the TGN has been suggested for AtRMR1–2 proteins (Occhialini et al., 2016), as well as for AtNIP1.1 (Barozzi et al., 2019). If the TGN can be identified as an early endosome, it must be considered that the endocytic degradation pathway involves multivesicular bodies/late endosomes (MVBs/LE), which cross paths with transport to lytic vacuoles (Cui et al., 2018).

We recently hypothesized that post-Golgi compartments may be considered as TGNs matured with the diversified contribution of Golgi-independent transport routes: MVBs/LE would receive material from endocytosis, EMAC (Delgadillo et al., 2020) and ERMEC (De Caroli et al., 2021) would receive material directly from ER. As observed for LD, plant vacuoles receive material from both the ER and Golgi traffic.

## 5 Vacuolar remodeling in response to environmental stresses

Plant vacuoles are highly adaptive and can remodel extensively (**Figure 1J**) to cope with environmental challenges such as osmotic stress, nutrient deprivation, and pathogen attack. During salt stress, for example, vacuoles act as critical reservoirs for excess ions, like sodium, sequestering them away from the cytoplasm to prevent ion toxicity and preserve enzymatic activity (Tan et al., 2019). The trafficking of ion transporters to the vacuolar membrane is a SNARE-dependent process, with SYP22 and other SNARE partners facilitating the fusion of vesicles containing Na^+^/H^+^ antiporters, aquaporins, and other transport proteins essential for ion homeostasis (Cao et al., 2020; Heinze et al., 2020). Drought stress triggers vacuolar adaptation as well, where aquaporins, known as tonoplast intrinsic proteins (TIPs), mediate rapid water flux across the vacuolar membrane allowing vacuoles to adjust their volume and maintain cell turgor (Cui et al., 2020). This adaptability to osmotic changes mirrors processes in adipocytes, where SNARE proteins regulate lipid droplet fusion and lipid homeostasis based on metabolic demands (Fu et al., 2023). In plants, the reconfiguration of vacuolar content and volume during stress highlights the crucial role of SNARE-mediated trafficking in cellular resilience.

In *Arabidopsis*, for example, SNARE proteins, like AtSYP121 and AtVAMP721/722, play key roles in both biotic (such as pathogen defense) and abiotic (including drought, salt, and osmotic stress) responses (Kwon et al., 2020). During biotic stress, these SNARE proteins support immune responses by guiding vesicles carrying defense molecules to sites of pathogen entry. In the face of abiotic stress, SNARE complexes help maintain cellular stability and function under harsh environmental conditions. Overall, the extensive diversity of SNARE proteins likely represents an evolutionary adaptation, allowing *Arabidopsis* to finely regulate stress responses and thrive despite its immobility in the environment.

## 6 Plant lipid droplets

Plant lipid droplets represent a subcellular compartment with strong analogies with adipocyte vacuoles. They originate from the ER, and their membrane is a phospholipidic monolayer. Inside or associated to this membrane, different proteins are present (oleosin, caleosin, steroleosin, seipin, lipid droplet-associated protein, and lipid droplet-associated protein-interacting protein) and are involved in their growth and stabilization (Cai and Horn, 2024). The presence of oleosin is important to avoid coalescence of the lipid droplet, and its presence allows lipid droplets to be referred to as oleosomes (Leprince et al., 1997; Cai and Horn, 2024). Only in plant or tissues defective for oleosin expression is it possible to see the spontaneous fusion of the lipid droplet (Deslandes et al., 2016). The main function of oleosomes is to provide an appropriate amount of energy especially during seed germination. The seed LDs are the most studied ones, but they are also found in other tissue of the plant where they are involved in important membrane remodeling processes related to pollen germination (Zienkiewicz et al., 2013; Krawczyk et al., 2022) or to heat, cold, and drought stresses (Gidda et al., 2016; Kim et al., 2016; Fernández-Santos et al., 2020).

LDs are also accumulated in the mesocarp of some fruits, like avocado (*Persea americana*), olive (*Olea europaea*), oil palm fruit (*Elaeis guineensis*), tung tree fruit (*Vernicia fordii*), and some sweet tropical fruits (Huang, 2018). The LDs accumulated in the pulp of these fruits lack oleosins and are larger (up to 20 µm) than those in seeds (up to 1 µm) (Huang and Huang, 2016). Avocado fruit pulp LDs are the most studied. In avocado fruit mesocarp, there are two different types of cells that differ in lipid composition. Fat cells are principally composed of LDs that accumulate saturated TAGs, while oil cells contain LDs rich in terpenoids. In fat cells (**Figure 1G**), it is possible to observe one to several large LDs (5–20 µm in diameter) and abundant small LD’s (0.1–0.2 µm in diameter). In the oil cells, a unique LD (also called oil drop) is present that occupies most of the cell volume (Huang and Huang, 2016). To confirm the absence of oleosin from LDs growing in size, it is interesting to note that transiently expressed oleosins fused to the fluorescent GFP tag in tobacco leaf protoplasts cannot be observed to label all LDs present in the cell but only the smallest ones (De Domenico et al., 2011).

If the process that describes LD formation is clear, the mechanism that brings LDs to fuse and increase in size is still unknown. A hypothesis could be that the small LDs, coated with oleosin, fuse with enlarging LDs, and after the fusion, the oleosin is not retained on the membrane (Huang, 2018), but this hypothesis does not explain the mechanism of the fusion. Structurally, SNAREs may play a role, but the only SNARE known to be involved in the formation of the LDs is the VAMP27-1, which interacts with seipin to stabilize the droplet formation on the ER but is then retained on the ER (Cai and Horn, 2024).

## 7 Transkingdom homologies of vacuolar SNAREs

The lysosome is a crucial vacuole in mammalian cells, and the SNARE complex involved in its formation is composed of STX7, STX8, VTI1B, and VAMP7 (Emperador-Melero et al., 2019). The biogenesis of melanosomes is facilitated by STX3, STX12/13, SNAP23, and VAMP7 (Ohbayashi and Fukuda, 2018). The formation of the adipocyte vacuoles is controlled by SNARE STX5, STX18, SNAP23, SEC22B, and VAMP4 (Boström et al., 2007; Fu et al., 2023). Would homologies between these genes and the plant genes provide new hints in the study of vacuoles?

The model plant *Arabidopsis thaliana*’s genes, corresponding to human SNARE homologs, were identified using the BLAST program (**Table 1**). Owing to the evolutionary gap between *Arabidopsis* and humans as well as the redundancy in the SNARE structure, the algorithm occasionally returns many homologs in *A. thaliana* for a single human SNARE. This investigation shows that the homologs *A. thaliana* SNAREs and human SNAREs share the same SNARE type (Qa, Qb, Qc, etc.) and that their localization is likewise preserved and, in certain cases, can aid in determining a more favorable homologous correspondence.

**Table 1:** List of human SNAREs involved in LRO formation, associated to their plant homologs.

*Homo sapiens*					*Arabidopsis thaliana*		
Involved in	SNARE	Type	Localization	References	SNARE	Type	Localization
Lysosome	STX7	Qa	EE	Emperador-Melero et al., 2019	SYP21	Qa	Vacuole/Golgy/cytosol
					SYP22		Vacuole
					SYP23		PM
	VTI1B	Qb	Lysosome/endosome		VTI12	Qb	Golgy/PM/vacuole
					VTI13		Golgy/vacuole/PM
	STX8	Qc	Golgy/endosome		SYP51	Qc	Vacuole
					SYP52		Vacuole
Melanosome	STX3	Qa	PM	Ohbayashi and Fukuda, 2018	SYP123	Qa	PM
					SYP131		PM
	STX13/12	Qa	Golgy/endosome		SYP22	Qa	Vacuole
					SYP23		PM
Melanosome/lysosome	VAMP7	R	LE/lysosome/PM/Golgy	Ohbayashi and Fukuda, 2018 Emperador-Melero et al., 2019	VAMP711	R	Vacuole/PM
					VAMP712		PM/vacuole/ER/Golgy
					VAMP713		Vacuole
					VAMP714		Golgy/PM/vacuole
Adipocyte's vacuoles	STX5	Qa	ER/Golgy	Boström et al., 2007 Fu et al., 2023	SYP31	Qa	Cytosol
					SYP32		Golgy
	STX18	Qa	ER		SYP81	Qa	Nucleus/ER/PM
	SEC22B	R	ER/Golgi		SEC221	R	RE/Golgy
	VAMP4	R	Golgy/PM/TGN		VAMP713	R	Vacuole
					VAMP714		Golgy/PM/vacuole
					VAMP727		Cytosol
Adipocyte's vacuoles/melanosome	SNAP23	Qb+Qc	PM/phagosome	Boström et al., 2007 Ohbayashi and Fukuda, 2018 Fu et al., 2023	SNAP29	Qb+Qc	Cytosol
					SNAP30		Cytosol
					SNAP33		PM/cytosol

Human protein localization data were obtained from the Uniprot database (https://www.uniprot.org/uniprotkb), whereas plant protein localization data were obtained from the ePlant database (https://bar.utoronto.ca/eplant/). The references in the table exclusively describe the individual human SNARE complex.

The lysosomal complex composed of VTI1B, STX7, STX8, and VAMP7 mediates late endosome–lysosome trafficking as well as the formation of autophagosomes (Emperador-Melero et al., 2019). The homologous SNAREs identified in *A. thaliana* are involved in the formation of the fusogenic complex to vacuoles. Core members of this complex include SYP21, SYP22, SYP51, SYP52, VTI12, and VTI13 (Sanderfoot et al., 2001), with the probable involvement of VAMP711/713/714 (Fujiwara et al., 2014).

According to Ohbayashi and Fukuda (2018), the creation and maturation of melanosomes are initiated by a material that is obtained by early endosomes and involves the following two distinct SNARE complexes: one consisting of STX3, VAMP7, and SNAP23, and another produced by STX13(12), VAMP7, and an unidentified Qb+Qc SNARE. The homologs of VAMP7 and SNAP23 in *A. thaliana* have been predicted to interact, but no interaction is yet demonstrated. This is not the case with the homologs of VAMP7 (VAMP711/712/713/714) and STX13 (SYP22/23) where the interactions between SYP22 and VAMP711/713/714 are known. The interactions between SYP22 and VAMP713 or VAMP714 need to be studied in more detail, but the interaction between SYP22 and VAMP711 was shown to form a complex with VTI11 and the SYP5 playing an important role in vesicle fusion to the vacuole (Fujiwara et al., 2014).

Concerning the formation of the adipocyte vacuoles, it is known that STX18, SEC22B, and SNAP33 interact and induce LD fusion (Fu et al., 2023). In contrast, *A. thaliana* homologs SYP81, SEC22, and SNAP29/30/33 do not exhibit any known interactions that result in the formation of a complex involved in LDs fusion; the results of interactions between these SNAREs are only obtained through the use of prediction tools such as AraNet, ePlant, and geneMANIA. Also, STX5, VAMP4, and SNAP23 form a complex involved in LD fusion (Boström et al., 2007). *A. thaliana* homologs SYP31/32, VAMP713/714/727, and SNAP29/30/33 were not yet found to interact, but the prediction software, again produces unfavorable outcomes. The unique SNARE protein, actually known to be involved in the formation of the LDs in plants, is the VAMP27–1 that interacts with the seipin to stabilize the droplet formation on the ER but is then retained on the ER (Cai and Horn, 2024).

A few works focus on the mechanisms of plant LD fusion. In the alga *Dunaliella bardawil*, LD proteosome analysis highlights the presence of Rab GTPases and of the membrane protein vesicle-inducing protein in plastids 1/inner membrane 30 (VIPP1/IM30) involved in vesicle formation and membrane fusion (Kroll et al., 2001; Davidi et al., 2014; Heidrich et al., 2016; Thurotte and Schneider, 2019). Another study on avocado fruit shows the presence of Rab GTPases and VAMP725 in the proteome of LDs recovered from the fruit pulp (Horn et al., 2013).

## 8 Advantages of a transkingdom approach for the study of vacuoles

By focusing on the SNARE gene family, we try to evidence analogies between animal and plant vacuoles. These compartments attract much interest among plant biologists because of their central role in the plant cell developmental strategy but stimulate less interest in the community of animal cell biologists because they are characteristic of specific cell kinds.

Of course, animal vacuoles, better known as LROs, are very important in the cells where they develop. The study of the related sorting mechanisms in some cellular models, such as melanosome maturation in melanocytes or LD fusion in adipocytes, serves as a model for the mechanisms shared by other cells developing LROs that are more difficult to investigate. Examples of these cells are the platelet dense granules, lung alveolar type II cell lamellar bodies (Bowman et al., 2019; Delevoye et al., 2019), immune system (Watts, 2022), and more. Naturally, animal cells are very diverse. Understanding the cellular machineries of melanosomes looking at the function of APs, BLOCs, and SNAREs is complicated by the ubiquitous expression and multiple roles of these components. For example, BLOC-1 in mouse cell lines is required to deliver cargoes from endosomes to the primary cilium (Monis et al., 2017), while in HeLa cells, BLOC-1 is required for recycling endosome morphogenesis (Delevoye et al., 2016). This is not surprising. In plants, where trafficking pathways differentiate more within a single cell, we observe a proliferation of genes within the same family, with overlapping and co-existing specific functions, and this may help to understand the functional variability offered by a single protein (Sanderfoot, 2007). Vacuolization aberrations can be seen in brain tumor cells incurring in extensive lipidization (Gaur et al., 2016), in balloon cell nevi forming benign melanocytic tumors occurring in conjunctiva, choroid, and skin, sebaceous adenomas, or metastatic renal carcinoma cells. The full comprehension of vacuole biology is then clinically significant (Thompson et al., 2015).

The important role of the SNARE complexes involved in the endocytic and secretory pathways is demonstrated by the fact that knockdown of a given SNARE dramatically alters specific transport events (Hong, 2005; Bethani et al., 2009). For instance, deletion of the SNARE proteins VAMP8 and Vti1b and STX17 impairs the autophagic process blocking the fusion of autophagosomes and xenophagosomes with lysosomes (Furuta et al., 2010; Itakura et al., 2012). Also, depletion of SNAP-25 and synaptobrevin perturbs endocytosis and exocytosis (Schoch et al., 2001; Washbourne et al., 2002; Zhang et al., 2013). Clearly, alterations of endocytosis and/or exocytosis affect many other different cellular processes. For instance, even neurite outgrowth is inhibited by siRNA-mediated knockdown of STX3 or by treatment of the dorsal root ganglion neurons with botulinum neurotoxin C, which inactivates STX3 (Schiavo et al., 1995; Igarashi et al., 1996; Darios and Davletov, 2006).

It was shown that SNAREs, through the regulation of transport pathways and signaling, are also involved in tumorigenesis (Meng and Wang, 2015). VAMP8 is a multifunctional R-SNARE (Antonin et al., 2000; Behrendorff et al., 2011; Itakura et al., 2012; Zhu et al., 2012) overexpressed in human glioma, a common brain tumor. It stimulates cellular proliferation *in vivo* and *in vitro* and promotes resistance to temozolomide (a chemotherapeutic drug used for the treatment of gliomas) enhancing autophagy (Chen et al., 2015). STX6 regulates endocytic recycling and affects chemotactic and cancer cell migration (Tiwari et al., 2011; Riggs et al., 2012). Furthermore, it influences cisplatin export in human ovarian cancer cell lines and contributes to chemoresistance (Moreno-Smith et al., 2013). These data suggest that modulation of the levels of expression of proteins, like VAMP8 and STX6, is important to precisely control intracellular traffic.

Overexpression of specific SNAREs may give rise to the phenomenon described by Varlamov and co-workers with inhibitory SNAREs (i-SNAREs). Studies in non-mammalian models can help in identifying the potential i-SNARE effect. In yeast, this effect can be observed in the Qc-SNAREs that control the competition between endosomal (Tlg1 and Syn8) and vacuolar forms (Vam7) of SNAREs (Izawa et al., 2012). They are also able to interact with V-ATPase subunits, and in doing so, they may change membrane traffic by influencing membrane potential (Strasser et al., 2011). The number of proteins potentially able to interact with SNAREs and change membrane potential is increasing (Grefen et al., 2010; Hachez et al., 2014). The characterization of Qc-SNAREs SYP51/52 in the model plant *Arabidopsis*, with the double localization on TGN and tonoplast, associated to two different functions (t-SNARE on TGN and i-SNARE on tonoplast) further expands the regulatory roles of SNAREs (De Benedictis et al., 2013). AtSYP51 in particular, homolog to the animal i-SNARE STX8 (Bilan et al., 2004), was suggested to be the key element of a complex regulatory mechanisms controlling the proportion of traffic from the Golgi (De Benedictis et al., 2013) compared to direct traffic from the ER. This direct traffic bypasses the Golgi traffic and is more dependent on microtubules than on actin transiting through the newly discovered compartment ERMEC (De Caroli et al., 2021). Interestingly, LD volume increase through fusion also depends on intact microtubule activity (Boström et al., 2007). Also, SYP21 shows i-SNARE proprieties, disturbing the transport from the PVC to LV (Foresti et al., 2006).

A few SNAREs can be clearly identified as i-SNARE (Di Sansebastiano, 2013) but an interesting implication of the i-SNARE effect can have an important implication also for human diseases. For instance, overexpression of STX1 inhibits exocytosis in hippocampal neurons (Mitchell and Ryan, 2005), while in pancreatic islets and insulinoma cell lines, overexpression of STX1 and STX3 proteins inhibits the biosynthesis and secretion of insulin and reduces the activity of the L-type Ca^2+^ channel (Kang et al., 2002). Overexpression of STX3 in MDCK cells inhibits transport from the TGN to apical plasma membrane and the endocytic recycling from the apical endosomes (Low et al., 1998). In addition, STX3 overexpression causes an accumulation of vesicles near the apical plasma membrane and blocking in vesicle fusion (Low et al., 1998).

STX5, found to be important in adipocyte vacuole formation (Boström et al., 2007), is also involved in the pre-Golgi and Golgi apparatus formation (Suga et al., 2005a). Its overexpression in BHK-21 cells inhibits ER to Golgi transport (Dascher et al., 1994). Additionally, the overexpression of STX5 induces the accumulation of βAPP (β-amyloid precursor protein) in the ER and cis-Golgi compartments and a reduction in Aβ peptide (β-amyloid peptide) secretion (Suga et al., 2005b). These data suggest that, probably, when overexpressed, STX5 interferes with the βAPP and Aβ peptide traffic and secretion (Suga et al., 2005b).

Overexpression of STX8 inhibits activity and trafficking of the CFTR (cystic fibrosis transmembrane conductance regulator) channel, and in particular, when STX8 is overexpressed, the localization of the CFTR channel at the plasma membrane decreases (Bilan et al., 2004).

STX18 is involved in ER-mediated phagocytosis (Hatsuzawa et al., 2006) and regulates trafficking between the ER and Golgi (Hatsuzawa et al., 2000). In HeLa cells, overexpression of STX18 and of a mutant form of the protein lacking its N-terminal domain induces aggregation of the ER, disassembly of the ERGIC and cis-Golgi, and blockage of the transport from the ER to Golgi (Hatsuzawa et al., 2000). Interestingly, STX18 is overexpressed in breast carcinomas (Sørensen et al., 2015), MDA-MB-436 breast cancer cells (Mecham, 2004), and clinical breast tumor samples (Bassett et al., 2008). Furthermore, it was shown that in MCF-7 cells, overexpression of STX18 inhibits cellular growth (Bassett et al., 2008). Notably, some of these SNAREs—STX18 (Fu et al., 2023), STX5 (Boström et al., 2007), STX3 and STX13 (Ohbayashi and Fukuda, 2018) can also be involved in vacuole formation and maturation.

The conservation of SNARE-mediated trafficking mechanisms between plants and animals underscores the value of comparative approaches in understanding vacuolar biology. By examining how plant cells harness these conserved molecular pathways for vacuolar maintenance, storage, and stress responses, insights may be gained into similar mechanisms governing lysosome and LRO function in animals. Additionally, SNAREs involved in vacuolar fusion and membrane remodeling in plants could promote agricultural strategies aimed at enhancing stress resilience or nutrient storage capacity, especially under adverse environmental conditions (Pereira and Di Sansebastiano, 2021).

## 9 Conclusions

Observation of molecular and cellular processes in different eukaryotic cells gives the opportunity to see the range of different solutions and combinations offered by similar components. This study highlights the significance of SNARE-mediated trafficking in both plant and animal vacuoles emphasizing how these mechanisms play a fundamental role in organelle formation, maintenance, and function. The structural and functional similarities between plant vacuoles and lysosome-related organelles (LROs) in animals suggest that a comparative approach can be instrumental in uncovering conserved and specialized pathways across kingdoms. We invite the scientific community to approach the study of vacuoles and LROs to bridge the gap between plant and animal cell biology, as interdisciplinary research on vacuoles and LROs could lead to breakthroughs in understanding intracellular trafficking, storage, and degradation processes. Future studies employing cross-kingdom analyses of SNARE interactions and trafficking networks may provide novel perspectives on disease mechanisms, metabolic regulation, and even biotechnological applications in agriculture and medicine. By fostering collaboration across disciplines, we can enhance our comprehension of cellular compartmentalization and its evolutionary significance.
